# *ABCB1* expression is increased in human first trimester placenta from pregnant women classified as overweight or obese

**DOI:** 10.1038/s41598-023-31598-5

**Published:** 2023-03-30

**Authors:** Signe Justesen, Katrine Bilde, Rasmus H. Olesen, Lars H. Pedersen, Erik Ernst, Agnete Larsen

**Affiliations:** 1 Department of Biomedicine, Aarhus University, Høegh-Guldbergs Gade 10, 8000 Aarhus C, Denmark; 2 Department of Obstetrics and Gynecology, Randers Regional Hospital, 8930 Randers, Denmark; 3 Department of Clinical Medicine, Aarhus University, 8200 Aarhus N, Denmark; 4 Department of Obstetrics and Gynecology, Aarhus University Hospital, 8200 Aarhus N, Denmark; 5 Department of Obstetrics and Gynecology, Horsens Regional Hospital, 8700 Horsens, Denmark

**Keywords:** Obesity, Reverse transcription polymerase chain reaction, Western blot

## Abstract

Obesity has become a global health challenge also affecting reproductive health. In pregnant women, obesity increases the risk of complications such as preterm birth, macrosomia, gestational diabetes, and preeclampsia. Moreover, obesity is associated with long-term adverse effects for the offspring, including increased risk of cardiovascular and metabolic diseases and neurodevelopmental difficulties. The underlying mechanisms are far from understood, but placental function is essential for pregnancy outcome. Transporter proteins P-glycoprotein (P-gp) and Breast Cancer Resistance Protein (BCRP) are important for trans-placental transport of endogenous substances like lipids and cortisol, a key hormone in tissue maturation. They also hold a protective function protecting the fetus from xenobiotics (e.g. pharmaceuticals). Animal studies suggest that maternal nutritional status can affect expression of placental transporters, but little is known about the effect on the human placenta, especially in early pregnancy. Here, we investigated if overweight and obesity in pregnant women altered mRNA expression of *ABCB1* encoding P-gp or *ABCG2* encoding BCRP in first trimester human placenta. With informed consent, 75 first trimester placental samples were obtained from women voluntarily seeking surgical abortion (< gestational week 12) (approval no.: 20060063). Villous samples (average gestational age 9.35 weeks) were used for qPCR analysis. For a subset (n = 38), additional villi were snap-frozen for protein analysis. Maternal BMI was defined at the time of termination of pregnancy. Compared to women with BMI 18.5–24.9 kg/m^2^ (n = 34), *ABCB1* mRNA expression was significantly increased in placenta samples from women classified as overweight (BMI 25–29.9 kg/m^2^, n = 18) (*p* = 0.040) and women classified as obese (BMI ≥ 30 kg/m^2^, n = 23) (*p* = 0.003). Albeit P-gp expression did not show statistically significant difference between groups, the effect of increasing BMI was the same in male and female pregnancies. To investigate if the P-gp increase was compensated, we determined the expression of *ABCG2* which was unaffected by maternal obesity (*p* = 0.291). Maternal BMI affects *ABCB1* but not *ABCG2* mRNA expression in first trimester human placenta. Further studies of early placental function are needed to understand how the expression of placental transport proteins is regulated by maternal factors such as nutritional status and determine the potential consequences for placental–fetal interaction.

## Introduction

The increasing prevalence of obesity among women in the reproductive age has become a major obstetric concern affecting millions of pregnant women worldwide^1–3^. Maternal obesity increases the risk of a plethora of pregnancy complications, including gestational diabetes, preeclampsia, preterm birth, macrosomia, and intrauterine death^4–8^. Numerous studies link maternal obesity to congenital malformations and long-term health problems for the children^1,9–15^, underlining that maternal weight has a significant influence on the intrauterine environment. These long-term effects include neurodevelopmental difficulties such as increased risk of autism spectrum disorders and metabolic problems such as obesity and type 2 diabetes^9,12,15^.

The connection between mother and child through the placenta is essential for fetal development, but because of this connection, the growing fetus is also susceptible to maternal exposures^16^. The apical membrane of the syncytiotrophoblast in the human placenta constitutes a protective barrier toward the fetus, a barrier which also regulates the influx of nutrition and growth factors, especially in early pregnancy. The efflux transporters P-glycoprotein (P-gp), encoded by the gene *ABCB1*, and Breast Cancer Resistance Protein (BCRP), encoded by the *ABCG2* gene, play an important role in the syncytiotrophoblast barrier function as they affect the transport of endogenous substances including sugars, amino acids, lipids, and steroids such as cortisol^17–20^. Both membrane proteins also have a major impact on pharmaco/toxicokinetics in the placenta transporting drugs and other xenobiotics away from the fetus^17,18,21^. A comparative study by Mathias and co-workers^22^ describing the mRNA and protein expression of *ABCB1*/P-gp and *ABCG2*/BCRP across pregnancy found that *ABCB1* mRNA and P-gp protein expression were significantly higher in first trimester compared to term placental tissue, a finding supported by other studies^23–25^. On the other hand, results on *ABCG2*/BCRP are not unambiguous, as Mathias et al. found no significant correlation between *ABCG2*/BCRP expression and gestational age^22^, whereas others have reported both a decrease and an increase with increasing gestational age ^23,25^.

Overall, a knowledge gap exists concerning placental transport function, especially in early pregnancy, albeit the high expression of P-gp in the first trimester could indicate that P-gp is of high importance at this time point. Guarding the growing fetus from harmful substances can be difficult, especially in cases where pharmacotherapy of the mother is necessary during organogenesis due to acute or chronic conditions^26^. P-gp and BCRP expression are known to be sensitive towards pharmacological exposures and can be both increased and reduced by pharmaceutical compounds. Use of P-gp and BCRP substrates are common in pregnancy. For example, in multi-morbid pregnancies, where pregnant women are treated with more than one type of medication, exposure to P-gp substrates has been reported to increase the risk of congenital malformations^27,28^, indicating that such changes are important for fetal development. Whether other factors besides pharmacological interventions can further affect P-gp and BCRP regulation is far from fully understood. In a rodent high-fat diet model of obesity, placental *Abcb1a* mRNA expression was decreased in high-fat-fed pregnant mice compared to placental expression levels in both control and undernourished pregnant mice, whereas placental *Abcg2* mRNA expression was increased in high-fat-fed pregnant mice compared to undernourished pregnant mice^29^. In line with this, two human studies on third trimester placental tissue have found maternal obesity to be associated with a decreased placental *ABCB1* mRNA expression and an increased *ABCG2* mRNA expression, respectively^25,30^. Collectively, these studies lead us to hypothesize that maternal nutritional status could affect the placental expression level of important efflux transporters. However, despite the increasing prevalence of overweight and obesity among women in the reproductive age, our knowledge of the impact of maternal nutritional status in first trimester human placenta is still sparse.

The aim of this cross-sectional study was therefore to compare *ABCB1* and *ABCG2* expression in first trimester placental tissue obtained from normal weight pregnancies (as defined by the current WHO criteria, BMI 18.5–24.9 kg/m^2^) with the expression in first trimester placental tissue obtained from pregnancies where the pregnant women could be classified as overweight (BMI 25–29.9 kg/m^2^) or as obese (BMI ≥ 30 kg/m^2^) according to the WHO.

## Materials and methods

### Ethical approval for collection of human placenta tissue

In this descriptive cross-sectional study, placental tissue was obtained from a Danish Regional Hospital (Central Denmark Region), donated by women who on their own accord chose to seek a surgical termination during the first trimester of pregnancy (before gestational week 12). Samples were collected with informed consent and with permission from The Danish National Committee on Health Research Ethics (approval no.:20060063), from women fulfilling the inclusion criteria; age 18 years minimum and with the ability to understand Danish. In addition to tissue donation, smoking habits and medication intake were noted based on self-reported questionnaires. All experiments were performed in accordance with relevant regulations and guidelines.

### Tissue collection

Immediately after the surgical abortion procedure, tissue was handled in sterilized physiological saltwater, and samples of free placental villi were collected. Tissue samples were stored in RNA later at − 20 °C before RNA extraction. If sufficient material was available, additional samples were snap frozen and stored at − 80 °C before protein extraction. From one donor, an additional sample of villous tissue was collected for immunohistochemistry and prepared by fixation in 0.1 M cacodylate buffer including 4% formaldehyde (pH 7.4) before dehydration in alcohol and embedding in paraffin.

### Selection of samples for the analysis

Maternal body mass index (BMI) was defined at the time of termination of pregnancy, and women were divided into three BMI groups according to the WHO guidelines: BMI 18.5–24.9 kg/m^2^ (normal weight), BMI 25–29.9 kg/m^2^ (overweight), and BMI ≥ 30 kg/m^2^ (obese). Women with pre-existing diabetes were excluded. Data on smoking habits and medication intake were based on self-reported questionnaires. A total of 75 samples were available for mRNA analysis, with an additional sample available for protein analysis in a subset of 38 samples. Only samples from singleton pregnancies were selected for analysis.

### RNA extraction and qPCR of synthesized cDNA

Total RNA was homogenized from up to 50 mg of frozen placental villi tissue by Trizol (Cat. No. 15596018, Ambion, Life Technologies, Denmark), extracted and purified using MaxTract high-density tubes (Cat. No 129056, Qiagen, USA) and RNeasy mini kit (Cat. No. 74104, Qiagen, USA) according to the manufacturer’s instructions. Homogenized samples were added to mini spin columns, and traces of genomic DNA were removed by DNase digestion with RNase-free DNase set (Cat. No. 79254, Qiagen, USA). RNA concentration and purity were determined by using PicoDrop instrument (PicoDrop Ltd, Cambridge, UK). cDNA was synthesized from 900 ng placental RNA using ImProm™ Reverse Transcription System kit (Cat. No. A2800, Promega, Denmark). Quantification of synthesized cDNA was performed by qPCR on an iCycler Thermal Cycler instrument (Bio-Rad, USA). Experiments were performed in duplicates in the presence of 4 µL 5 × HOT FIREPol EvaGreen® qPCR Mix Plus (ROX) (Cat. No. 08-24-00020, Taq Copenhagen, Denmark), 2 µM primers and 15 ng cDNA in a final volume of 25 µL. Cyclophilin A (*CYPA*) and hypoxanthine–guanine phosphoribosyltransferase (*HPRT*) were used as reference genes. Stability of reference genes were confirmed by geNorm software (geNorm 3.5, Microsoft Excel). All primer sequences are listed in Table 1.

**Table 1:** Primer sequences used for analysis.

Gene name	Forward primer	Reverse primer	Tm (˚C)
*ABCB1*	5′ CAGGGAAAGTGCTGCTTGATG 3′	5′ TCGATGAAGGCATGTATGTTGG 3′	54
*ABCG2*	5′ CAGGTCAGAGTGTGGTTTCTG 3′	5′ TCTTCGCCAGTACATGTTGC 3′	54
*CYPA*	5′ GCCGAGGAAAACCGTGTACTA 3′	5′ ACCCTGACACATAAACCCTGG 3′	60
*HPRT*	5′ CCTGGCGTCGTGATTAGTGA 3′	5′ GAGCACACAGAGGGCTACAA 3′	59
*SRY*	5′ CATGAACGCATTCATCGTGTGGTC-3′	5′ CTGCGGGAAGCAAACTGCAATTCTT 3′	60

Primers were designed using the National Center for Biotechnology Information webpage (www.ncbi.nlm.nih.gov). Primer specificity was confirmed by NCBI Basic Local Alignment Search Tool.

### Determination of fetal sex

Following Danish law, fetal sex was determined in the laboratory, after completion of the abortion procedure, without knowing the identity of the donor and thereby without the possibility to report the result back to the donor. Fetal sex was determined by using a primer specific to the Sex-determining Region gene in the Y chromosome (SRY). For each sample, a PCR reaction was performed in an Eppendorf Master Cycler (Eppendorf AG, Hamburg, Germany) using HotStarTaq Master Mix (Cat. No. 203445, Qiagen), 1 µM primers and 3 ng placenta cDNA in a total volume of 10 µl. The presence or absence of the SRY gene was visualized on a 1% agarose gel. Primer sequences are listed in Table 1.

### Protein extraction and Western blotting

Villous tissue was homogenized in Pierce RIPA buffer (Cat. No. 89901, Thermo Scientific, Denmark) with protease and phosphatase inhibitor cocktail (Cat. No. 78440, Thermo Scientific, Denmark). Protein concentration was determined using Pierce™ BCA protein assay kit (Cat. No. 23227, Thermo Scientific, Denmark). Total protein of 10 µg was separated on precast polyacrylamide gels (4–12% XT Bis–Tris Protein Gel, Cat. No. 3450125, Bio-Rad, Denmark) and transferred to ethanol pre-soaked PDVF membranes. Prior to incubation with primary antibody, the membranes were cut horizontally to allow incubation with both target and loading control at the same time. The membranes were blocked in 5% skimmed milk in tris-buffered saline containing Tween20 (TBST) and incubated overnight at 4 °C with primary anti-P-glycoprotein antibody (1:4000 dilution, Cat. No. ab170904, Abcam, Denmark). After washing in TBST, the membranes were incubated with horseradish peroxidase-conjugated (HRP) secondary anti-rabbit antibody (1:4000 dilution, Cat. No. sc-2054, Santa Cruz, Germany), and protein-antibody complexes were visualized using chemiluminescence detection reagents (Clarity™ Western ECL Substrate, Cat. No. 1705060, Bio-Rad, Denmark). Protein levels were quantified with ImageQuant (Image Quant LAS 4000). Protein signals were reported relative to Cyclophilin B (CYPB) (Cat. No. ab178397, Abcam, Denmark), after statistically confirming stable expression of CYPB between relevant groups.

### Immunohistochemistry

Five µm thick paraffin-embedded sections of placental tissue were dewaxed and rehydrated before antigen retrieval. Endogenous tissue peroxidase activity was blocked in 3% hydrogen peroxide in ethanol, and permeability was obtained by 0.5% Triton X100 in PBS. Blocking was performed by using 10% normal goat serum (Cat. No. 10000C, Thermo Fisher, Life Technologies, Denmark) in PBS for 30 min. Sections were incubated overnight at 4 °C with primary anti-P-glycoprotein antibody (ab170904, Abcam, Denmark) in a 1:200 dilution in PBS containing 10% goat serum. A negative control was incubated in PBS containing 10% goat serum only. After washing, sections were incubated in 1:400 diluted HRP-conjugated goat anti-rabbit secondary antibody (Cat. No. 7074, Cell Signaling, Denmark). Sections were developed with DAB substrate solution (Dako, USA) and counterstained with hematoxylin. Images were captured by using Leica microscope (DMI400B) and Leica LAS software (Leica Microsystems, Limited Version 4.11.0, Switzerland).

### Statistical analyses

Based on qq-plots, expression data (whole dataset) were confirmed normally distributed. Prior to comparison between groups, a similar approach was used to confirm normality within each group. The association between gene expression and BMI or gestational age was studied using a linear regression. A one-way ANOVA was used to compare the gene expression level between the three BMI groups (normal BMI 18.5–24.9 kg/m^2^, overweight BMI 25–29.9 kg/m^2^, and obese BMI ≥ 30 kg/m^2^). Prior to performing ANOVA analysis, equal variance was tested using Bartlett’s test, and in cases with statistically significant unequal variance a Welch’s ANOVA was performed with unpaired t-test with Welch’s correction as post-hoc test, considering the normal weight group as the reference group. When comparing only two groups, i.e. smokers versus non-smokers and male versus female pregnancies, respectively, a Student’s t-test was used to compare gene expression. In cases of non-normal distributions a log transformation was performed, and ultimately a Mann–Whitney test was used, as data did not follow a normal distribution after the transformation. A *p* value < 0.05 was considered as a statistically significant difference in all analyses. For statistical analyses, GraphPad Prism version 9 (GraphPad Software, La Jolla, CA, USA) was used.

## Results

**Figure 1:**
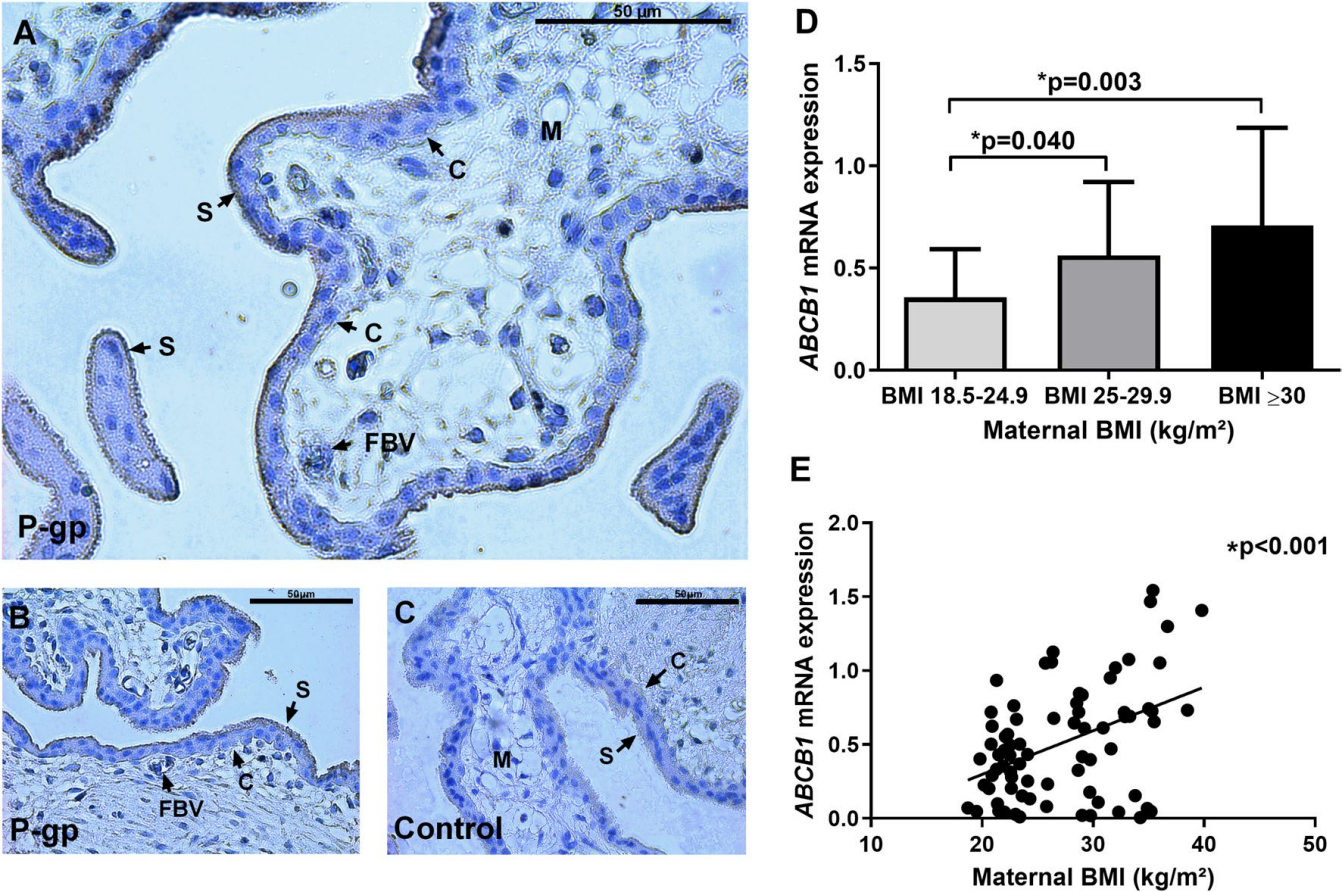
Localization of P-gp and *ABCB1* mRNA expression in placental tissue. (**A**,**B**) Immunohistochemistry image of placental tissue using antibody against P-glycoprotein (P-gp) and stained with H&DAB. (**C**) Negative control without primary antibody. Arrowheads denote basophilic cytotrophoblastic cells (C), fetal blood vessel (FVB), mesenchyme (M), syncytiotrophoblastic layer (S). Magnification × 40. Scalebar represents 50 µM. Localization of P-gp in the apical membrane of the syncytiotrophoblast was confirmed, in addition to some faint immunostaining visible in the syncytiotrophoblast basolateral membrane. (**D**) *ABCB1* mRNA expression in the three maternal BMI groups; normal weight (BMI: 18.5–24.9 kg/m^2^ (n = 34)), overweight (BMI: 25–29.9 kg/m^2^ (n = 18)), and obese (BMI ≥ 30 kg/m^2^ (n = 23)) (Welch’s ANOVA, W(2.000,33.800) = 6.555, *p* = 0.004, was used due to unequal variance (Bartlett’s test, *p* = 0.002)). Statistically significant difference between the normal weight group and the overweight group (95% CI [0.011, 0.401], *p* = 0.040) and between the normal weight group and the obese group (95% CI [0.130, 0.571], *p* = 0.003) (post-hoc test: unpaired t-test with Welch’s correction). A *p* value < 0.05 was considered statistically significant. Each bar represents mean ± standard deviation (SD). (**E**) Expression of *ABCB1* evaluated by linear regression. Each dot represents one sample (n = 75). Line represents best fitted line. Slope = 0.030, F(1,73) = 16.36, R^2^ = 0.183, *p* < 0.001. A *p* value < 0.05 was considered statistically significant.

Based on our selection criteria we were able to obtain 75 samples for qPCR and 38 samples for Western blot analysis. For both studies, a villous sample similar to Fig. 1A–C was used. Demographic details on the individual pregnancies are depicted in Table 2.

**Table 2:** Characteristics of first trimester placental samples used for mRNA and protein expression analysis.

	BMI 18.5–24.9	BMI 25–29.9	BMI ≥ 30	*p* value
Samples used in mRNA analysis				
Total (n)	34	18	23	
Maternal BMI (kg/m^2^)	21.99 (1.36)	28.05 (1.49)	34.19 (2.33)	< 0.0001
Gestational age (weeks)	9.46 (1.36)	9.48 (1.27)	9.11 (1.21)	0.541
Smokers (n (%))	14 (41.2)	8 (44.4)	8 (34.8))	
Medication intake (n (%))	2 (5.9)	0 (0)	2 (8.7)	
Fetal sex (♀/♂)	13/21	9/9	13/10	
Samples used in protein analysis				
Total (n)	14	10	14	
Maternal BMI (kg/m^2^)	22.13 (1.29)	28.58 (1.23)	34.13 (2.34)	< 0.0001
Gestational age (weeks)	10.02 (1.34)	9.48 (1.38)	8.68 (1.18)	0.032
Smokers (n (%))	2 (14.3)	4 (40.0)	3 (21.4)	
Medication intake (n (%))	2 (14.3)	0 (0)	2 (14.3)	
Fetal sex (♀/♂)	3/11	3/7	6/8	

Maternal BMI and gestational age are given as mean ± standard deviation (SD). One-way ANOVA was used to identify significant difference between groups.

BMI: body mass index.

### *ABCB1* mRNA expression was significantly increased in placental tissue from women classified as overweight or obese

Comparing the BMI groups, Welch’s ANOVA found a statistically significant difference in *ABCB1* mRNA expression (Welch’s ANOVA, W(2.000, 33.800) = 6.555, *p* = 0.004). Post-hoc tests found that, compared to the normal weight group (BMI 18.5–24.9 kg/m^2^), the *ABCB1* mRNA expression was increased 1.5-fold in placental tissue from women classified as overweight (95% CI [0.011, 0.399], *p* = 0.040) and twofold in placenta from women classified as obese (95% CI [0.130, 0.571], *p* = 0.003) (Fig. 1D), respectively. Correspondingly, regression analysis of all samples found a statistically significant linear relationship between increasing maternal BMI and increasing *ABCB1* mRNA expression throughout the cohort (R^2^ = 0.183, *p* < 0.001, Fig. 1E). This was also evident if looking at the BMI ≥ 35 kg/m^2^ group separately, compared to the normal weight group (95% CI [0.268, 0.952], *p* = 0.003, Supplementary Fig. S1).

**Figure 2:**
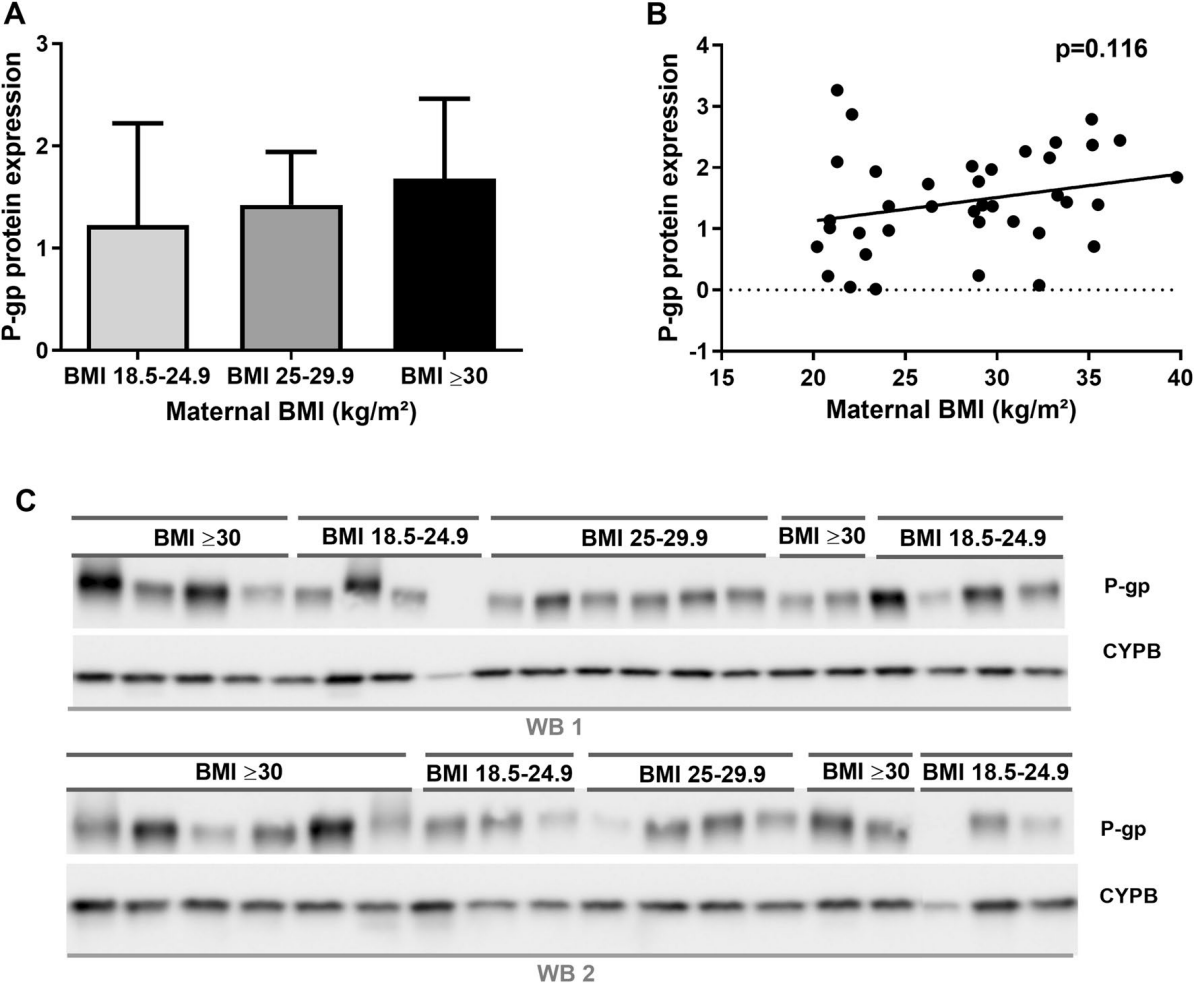
P-gp protein expression and maternal BMI in placental tissue. (**A**) P-gp protein expression in the three maternal BMI groups; normal weight (BMI: 18.5–24.9 kg/m^2^ (n = 14)), overweight (BMI: 25–29.9 kg/m^2^ (n = 10)), and obese (BMI ≥ 30 kg/m^2^ (n = 14)). No statistically significant difference was found (one-way ANOVA, F(2,35) = 1.080, *p* = 0.351). A *p* value < 0.05 was considered statistically significant. Each bar represents mean ± SD. (**B**) Protein expression of P-gp analyzed by linear regression. Each dot represents one sample (n = 38), and line represents best fitted line. Slope = 0.039, F(1,36) = 2.59, R^2^ = 0.067, *p* = 0.116. A *p* value < 0.05 was considered statistically significant. (**C**) Western blot of P-gp and loading control CYPB, cropped blots. Full blots available in Supplementary Fig. S3–S4.

Western blotting performed on the available subgroup (n = 38), consisting of placental tissue from women classified as normal weight (n = 14, average gestational age: 10.02 weeks), overweight (n = 10, average gestational age: 9.48 weeks), or obese (n = 14, average gestational age: 8.68 weeks), respectively, did not find any statistically significant differences in P-gp protein expression between the three BMI groups (One-way ANOVA, F(2,35) = 1.080, *p* = 0.351). Linear regression analysis revealed no statistically significant relationship between P-gp protein expression and maternal BMI (R^2^ = 0.067, *p* = 0.116) (Fig. 2A–C).

### Fetal sex did not affect *ABCB1* mRNA expression

**Figure 3:**
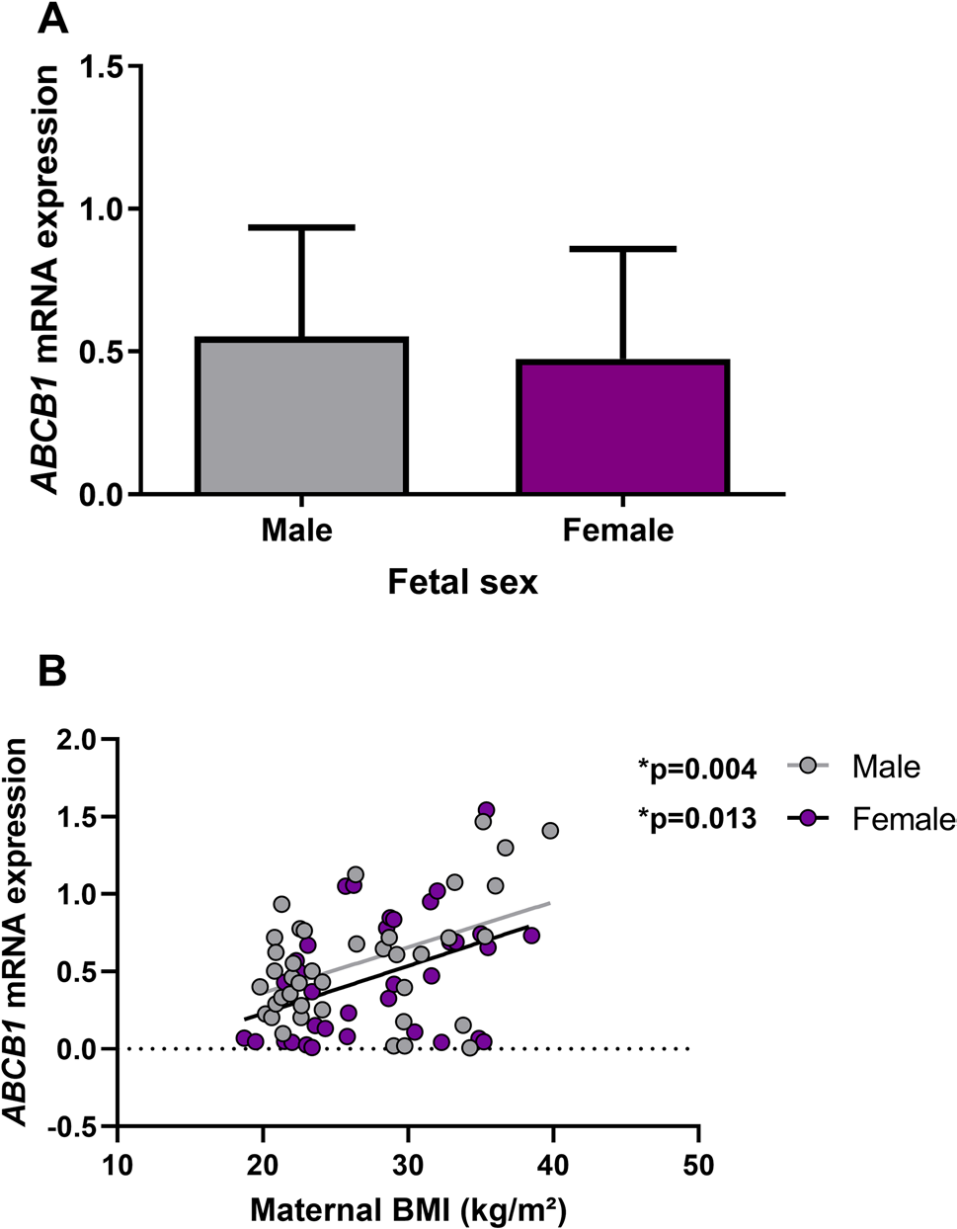
*ABCB1* mRNA expression in placental tissue in relation to fetal sex. (**A**) Expression of *ABCB1* in placental tissue between male (n = 40) and female (n = 35) fetuses. No significant difference between the two groups (Mann–Whitney test, Mann–Whitney *U* = 612, median = 0.503 (male) and median = 0.431 (female), *p* = 0.355). A *p* value < 0.05 was considered statistically significant. Each bar represents mean ± SD. (**B**) Expression of *ABCB1* between male and female fetuses evaluated by linear regression, (male: Slope = 0.029, F(1,38) = 9.480 R^2^ = 0.199, *p* = 0.004, female: Slope = 0.031, F(1,33) = 6.932 R^2^ = 0.174, *p* = 0.013). Each dot represents one sample (n = 75), and line represents best fitted line. A *p* value < 0.05 was considered statistically significant.

Comparing the mRNA expression in placental tissue discriminating between male (n = 40, average gestational age: 9.35 weeks) and female (n = 35, average gestational age: 9.38 weeks) fetuses, we found that the *ABCB1* expression was not affected by fetal sex (Mann–Whitney *U* = 612, median = 0.503 (male) and median = 0.431 (female), *p* = 0.355, Fig. 3A).

Additionally, linear regression analyses were run separately on placental tissue from male and female fetuses to see if the placental *ABCB1* response to increasing BMI was similar in male and female pregnancies. Here, we observed a statistically significant increase in *ABCB1* mRNA expression with increasing maternal BMI in both groups (male: R^2^ = 0.199, *p* = 0.004 and female: R^2^ = 0.174, *p* = 0.013 respectively, Fig. 3B).

### Increasing maternal BMI was not associated with changes in the placental *ABCG2* mRNA expression

**Figure 4:**
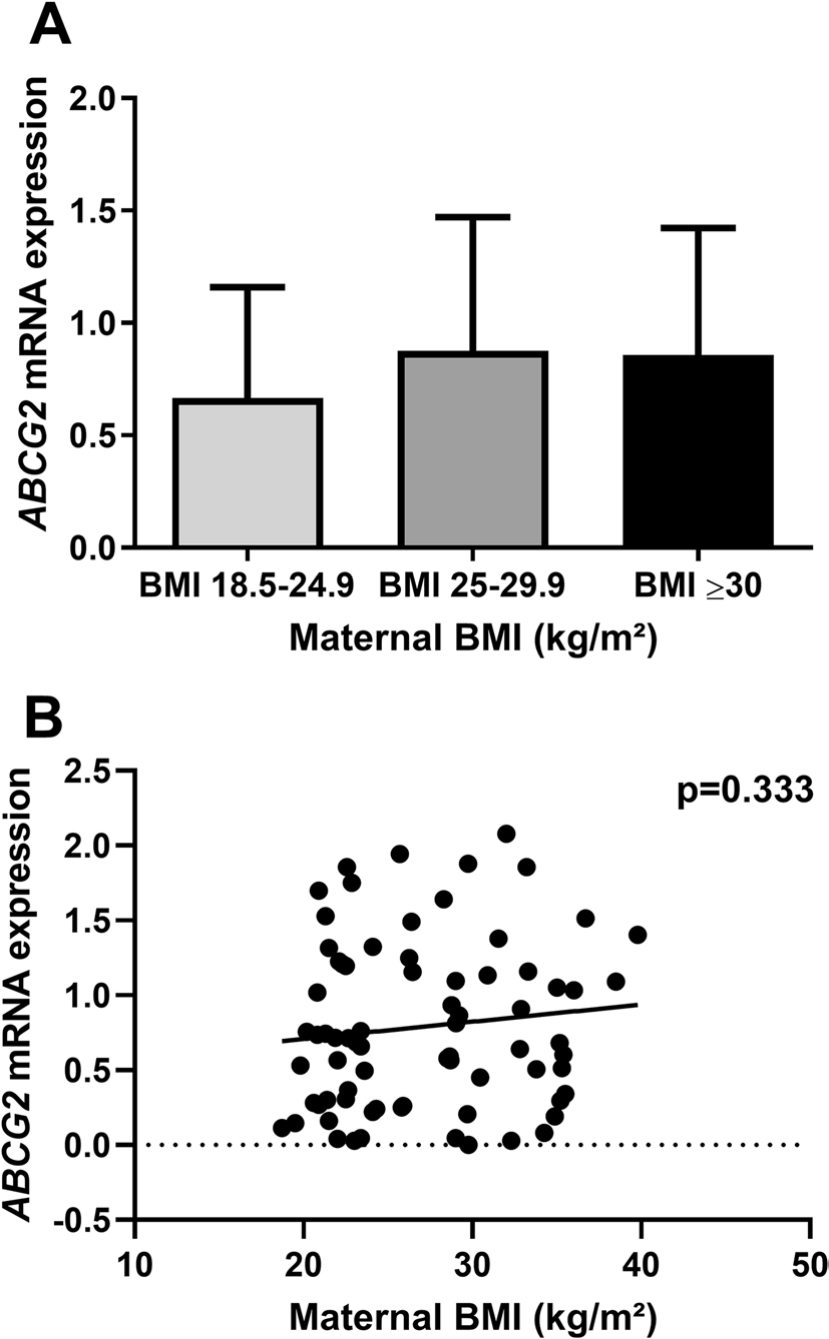
*ABCG2* mRNA expression and maternal BMI in placental tissue. (**A**) *ABCG2* mRNA expression in the three maternal BMI groups; normal weight (BMI: 18.5–24.9 kg/m^2^ (n = 34)), overweight (BMI: 25–29.9 kg/m^2^ (n = 18)), and obese (BMI ≥ 30 kg/m^2^ (n = 22)). No statistically significant difference was found (one-way ANOVA, F(2,71) = 1.255, *p* = 0.291). A *p* value < 0.05 was considered statistically significant. Each bar represents mean ± SD. (**B**) Expression of *ABCG2* evaluated by using linear regression. Each dot represents one sample (n = 74), and line represents best fitted line. Slope = 0.011, F(1,72) = 0.949, R^2^ = 0.013, *p* = 0.333. A *p* value < 0.05 was considered statistically significant. One sample was removed due to technical reasons.

As P-gp is not the only important efflux pump in the apical syncytium, we wanted to investigate if changes in *ABCB1* expression were counteracted or supported by changes in the other major efflux pump; BCRP (encoded by the *ABCG2* gene). No statistically significant difference was found in the placental *ABCG2* mRNA expression between the three BMI groups (One-way ANOVA, F(2,71) = 1.255, *p* = 0.291). Similarly, linear regression analysis showed no statistically significant linear relationship between placental *ABCG2* mRNA expression and maternal BMI (R^2^ = 0.013, *p* = 0.333) (Fig. 4A + B).

### Effect of gestational age and maternal smoking on *ABCB1* and *ABCG2*

As both *ABCB1/*P-gp and *ABCG2/*BCRP expression is known to be susceptible to xenobiotic exposures and at least P-gp can be affected by gestational age^22,24,31^, we further examined if differences in gestational age and maternal smoking status could have an impact on our findings. Investigating the whole dataset with gestational age from week 6 to 12 (average 9.35 weeks), we found a statistically significant inverse correlation between increasing gestational age and *ABCB1* mRNA expression (R^2^ = 0.06, *p* = 0.035, Supplementary Fig. S2). However, no statistically significant difference in mean gestational age between the three BMI groups was seen (One-way ANOVA, F(2,72) = 0.619, *p* = 0.541, Table 2). No significant correlation between gestational age and mRNA expression was found for *ABCG2* (R^2^ = 0.034, *p* = 0.116, data not shown). A fraction of the participants (n = 30) declared smoking on a regular basis. It has been debated if cigarette compounds could affect P-gp expression^31,32^, and we therefore tested if smoking affected the mRNA expression of *ABCB1* (95% CI [-0.106, 0.255], *p* = 0.414) or *ABCG2* (95% CI [-0.201, 0.356], *p* = 0.651), and we found no significant difference in BMI between the smoking (average BMI: 26.6 kg/m^2^) and the non-smoking groups (average BMI: 27.5 kg/m^2^). Self-reported use of medication was very limited (four in total – two women classified as normal weight and two classified as obese, reported some use of acetaminophen or antiacids), so no statistical test was performed.

## Discussion

In this study, we found a statistically significant increase in *ABCB1* mRNA expression with increasing maternal BMI indicating that obesity-induced placental changes occur as early as the first trimester, a sensitive time in human development where the effects of maternal BMI have not previously been examined. However, the fact that we cannot investigate the functional consequences of such *ABCB1* upregulation of course poses a limitation of the study. With only a limited sample size for Western blot, with varying gestational age, we saw no statistically significant alteration in the P-gp protein expression, and no studies of P-gp function could be performed on the samples collected here.

In contrast to this study, a previous study by Wang and co-workers found an obesity-induced decrease in P-gp expression in human term placenta^30^. Notably, slightly different BMI categories were used by Wang and colleagues, who defined obesity as women with pre-gravid BMI ≥ 28 kg/m^2^ by which the average BMI among women with obesity was 29.10 kg/m^2^ compared to 35.97 kg/m^2^ in our study. However, this difference in BMI categories is less likely to affect the results when one considers that ethnic differences has been found when using BMI to predict total body fat. It is thus known that Chinese individuals have lower BMI for the same body fat as Caucasians^33^. Interestingly, evidence suggests that maternal dyslipidemia during pregnancy affects the placental aging^34^, pointing towards a link between maternal pre-pregnancy obesity and placental aging. If premature placental ageing is seen in women classified as obese, this might lead to a more rapid decrease in P-gp expression towards late pregnancy. If so, this might be a reason why Wang and colleagues observe a decreased *ABCB1*/P-gp expression in term placenta from women classified as obese^30^. In a very recent study by Scott et al.^25^, *ABCG2* but not *ABCB1* expression was increased with increasing maternal BMI levels in third trimester human placenta. This is consistent with animal studies^29^, whereas this study did not find an effect of increasing maternal BMI on *ABCG2* levels. Interestingly, the authors also showed that inflammation associated with chorionamnionitis resulted in increased P-gp expression. This is in contrast to numerous in vitro studies finding that pro-inflammatory cytokines can cause down-regulation of P-gp in different cell types such as primary trophoblasts, brain endothelial cells, and hepatocytes^35–37^. In vivo however, obesity has shown to induce the hepatic expression of P-gp in rodents^38^ indicating a complex regulatory mechanism which might be tissue-dependent. It should also be considered that the study by Scott and co-workers^25^ investigated the expression of placental tissue exposed to numerous pharmacological substances. P-gp/*ABCB1* and BCRP/*ABCG2* expression is easily manipulated pharmacologically^39^, and difference in medication intake among the women could have an impact on placental expression. However, the self-reported prevalence of medication intake was limited among the women in this study as only reported by two women with normal weight and two women with obesity (equal to 5.3% of all the included women). A similar prevalence of exposure was found in blood samples from another cohort of first trimester pregnant Danish women, where traces of prescription drugs and over-the-counter medication were found in 5.3% and 8.9% of the women, respectively^40^, supporting that the self-reports reflect the actual exposure pattern. Overall, it is a strength of the present study, that the amount of pharmacological exposures are indeed very limited, hereby providing stronger data on the impact of maternal body composition on *ABCB1* and *ABCG2* expression.

If the observed *ABCB1* changes seen in this study are in fact accompanied by changes in the P-gp expression without any reduction in P-gp activity, this could potentially affect embryonic development in numerous ways. Among others, P-gp is important in cortisol regulation^41^ and lipid transportation^32^. Alterations in cortisol supply to the fetus could hence influence fetal organ and especially brain maturation^42,43^, an organ believed to be sensitive towards maternal obesity^10,14,15^. When it comes to lipid transportation, an up-regulation of P-gp could result in an increased amount of lipids reaching the developing placenta. It is well-known that obesity is associated with lipid abnormalities (e.g. increased levels of triacylglycerols and cholesterol^44^), and abnormal lipid metabolism is seen in placentas from women categorized as obese with an increase in placental lipid esterification and lipid storage^45^ underlining the potential adverse effects of obesity and altered lipid metabolism on the placental function.

If the increased blood lipids associated with Western diet and obesity^45^ are also directly involved in P-gp regulation, they should be targeted in future studies analyzing matching samples of placental tissue and maternal blood. Such studies should also investigate the possible relationship between maternal inflammation and placental P-gp expression. An obesity-associated increase in inflammatory markers has been observed in pregnant women as early as first trimester^46^ and could thus be another potential factor for regulation of *ABCB1*/P-gp expression, although current data from the literature is not consistent in determining if the most likely result is an up- or a down-regulation of *ABCB1*/P-gp and *ABCG2*/BCRP with increasing maternal inflammation.

As no blood samples were available, the possible correlation between P-gp and inflammation could not be investigated. Systemic inflammation as a consequence of regular cigarette smoking^47^ could however be a confounding factor, and we therefore tested if smoking affected the expression of *ABCB1* but found no significant difference in placental *ABCB1* mRNA expression between tissue from smoking (n = 30) and non-smoking (n = 45) women or when comparing tissue from women classified as normal weight, overweight or obese separately (data not shown).

Another possible confounder was gestational age, as an age-dependent decrease in *ABCB1* expression in first trimester was evident in our data. This is in line with other reports on human placenta during gestation week 6 to term^22,24^. One could speculate if the maternal obesity-associated difference is a consequence of difference in gestational age. However, in our study, when evaluating the gestational age in the different BMI groups, no difference was found between groups.

In conclusion, maternal obesity significantly increases the expression of *ABCB1* in first trimester placenta villous tissue. These findings indicate that obesity-induced placental changes occur as early as the first trimester and underlines the need to understand how maternal body composition and nutritional status affect the intra-uterine environment and fetal programming. The obesity-induced P-gp dysregulation in placenta needs further investigations to assess the need for interventions ameliorating obesity-induced placental dysfunction.

## Supplementary Information

Supplementary Information.

## Data Availability

The data underlying this article cannot be shared publicly due to the privacy of the individuals that participated in the study. The data will be shared on reasonable request to the corresponding author.

## Abbreviations

BCRP: Breast cancer resistance protein
BMI: Body mass index
CYPA: Cyclophilin A
CYPB: Cyclophilin B
HPRT: Hypoxanthine–guanine phosphoribosyltransferase
HRP: Horseradish peroxidase
P-gp: P-glycoprotein
SRY: Sex-determining Region Y
TBST: Tris-buffered saline containing Tween20
