# Supplementary material for: ABCB1 expression is increased in human first trimester placenta from pregnant women classified as overweight or obese
Source: Sci Rep. 2023 Mar 30;13:5175. doi: 10.1038/s41598-023-31598-5 (PMC10063677; doi:10.1038/s41598-023-31598-5)
Supplement: Supplementary file 1 — Supplementary Information. [file 41598_2023_31598_MOESM1_ESM.docx]

Supplementary Information

***ABCB1* expression is increased in human first trimester placenta from pregnant women**

**classified as overweight or obese**

**S. Justesen, K. Bilde, R.H. Olesen, L.H. Pedersen, E. Ernst, and A. Larsen**

**Contents**

Supplementary Figure S1. Placental *ABCB1* expression in relation to maternal BMI

Supplementary Figure S2. Gestational age and *ABCB1* mRNA expression

Supplementary Figure S3. Uncropped version of P-gp Western blot

Supplementary Figure S4. Uncropped version of CYPB Western blot

**Supplementary Figure S1. Placental *ABCB1* expression in relation to maternal BMI**


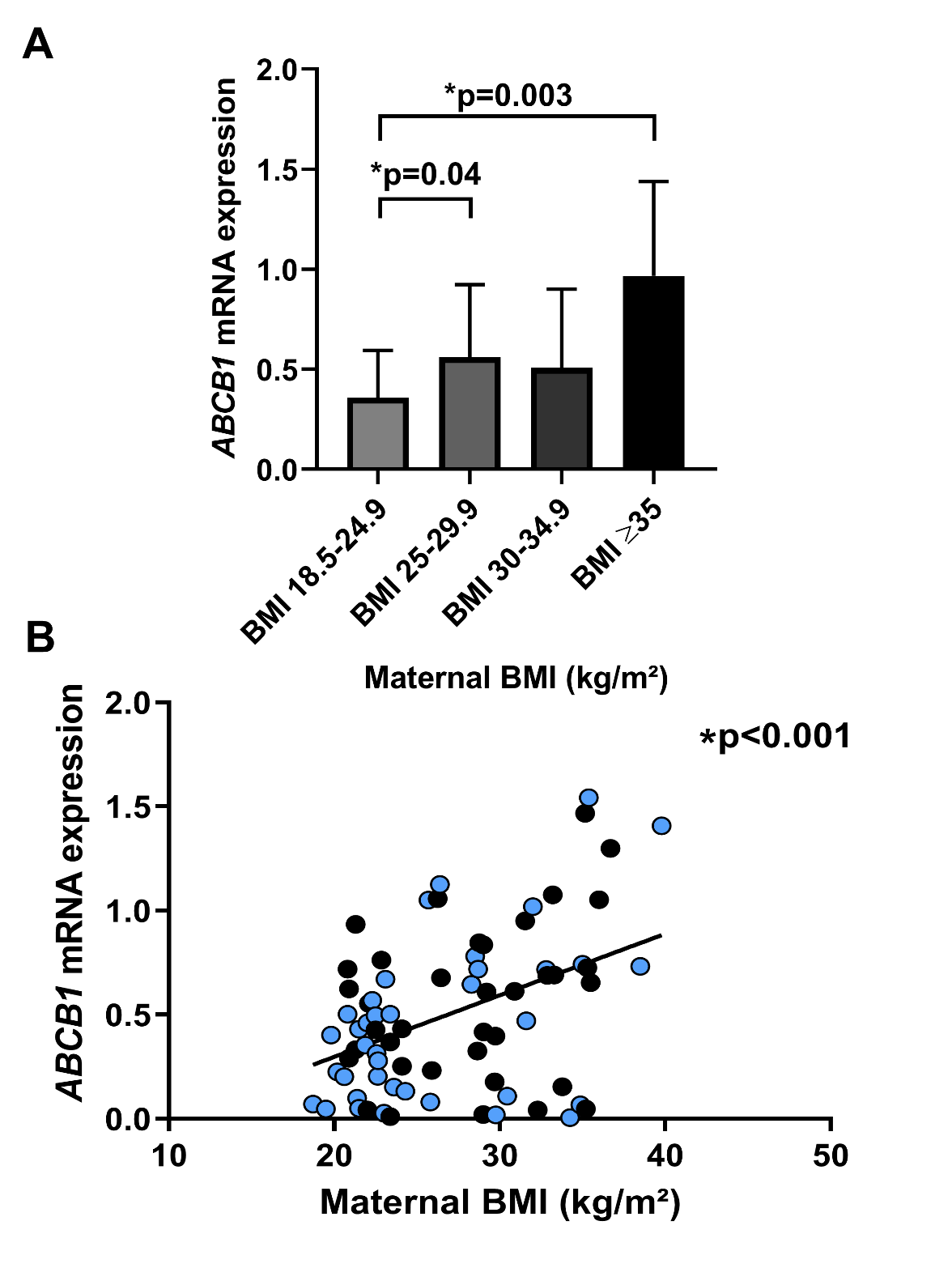


**Supplementary Figure S1**. **Placental *ABCB1* expression in relation to maternal BMI. A)** *ABCB1* mRNA expression between maternal BMI groups (Welch’s ANOVA, W(3.000,23.520)=6.030, p=0.003, post hoc test: unpaired t-test with Welch’s correction). Each bar represents mean ± standard deviation (SD). **B)** Expression of *ABCB1* evaluated by linear regression. Each dot represents one sample (n=75). Black dots represent samples from same placenta used for both protein- and mRNA analysis while blue dots represent samples only used in mRNA analysis. Line represents best fitted line. Slope=0.030, F(1,73)=16.36, R^2^=0.183, p<0.001. A p-value <0.05 was considered statistically significant.

**Supplementary Figure S2. Gestational age and *ABCB1* mRNA expression**

**Supplementary figure S2. Gestational age and *ABCB1* mRNA expression.** Effect of gestational age on mRNA expression of *ABCB1* analyzed by using linear regression. Each dot represents one sample (n=75) and line represents best fitted line. Slope=-0.07, F(1,73)=4.62, R^2^=0.06, p=0.035. A p-value <0.05 was considered statistically significant.

**Supplementary Figure S3. Uncropped version of P-gp Western blot**

**

**

**Western blot 1**

**kDa**

**250-**

**150-**

**100-**

**Western blot 2**

**kDa**

**250-**

**150-**

**100-**

**Supplementary Figure S3. Uncropped version of P-gp Western blot.** Protein expression of P-glycoprotein (P-gp) in placental tissue determined by Western blotting. Membranes from western blot 1 and 2 were cut horizontally prior to incubation with primary antibody allowing incubation with target and loading control (see supplementary figure 4) at the same time. Prior to quantification and statistical analyses, a total of four samples were removed (visible here but have been cropped in Figure 2). Three samples were removed due to technical reasons and one sample was removed as it was discovered it came from a multiple pregnancy.

**Supplementary Figure S4. Uncropped version of CYPB Western blot**





**Western blot 1**

**kDa**

**37-**

**25-**

**20-**

**Western blot 2**

**kDa**

**37-**

**25-**

**20-**

**Supplementary Figure S4. Uncropped version of CYPB Western blot.** Protein expression of cyclophilin B (CYPB), used as loading control, in placental tissue determined by Western blotting. Membranes from western blot 1 and 2 were cut horizontally prior to incubation with primary antibody allowing incubation with target (see supplementary figure 3) and loading control at the same time. Prior to quantification and statistical analyses, a total of four samples were removed (visible here but have been cropped in Figure 2). Three samples were removed due to technical reasons and one sample was removed as it was discovered it came from a multiple pregnancy.
